# The clustering status of detached gastric cancer cells inhibits anoikis-induced ferroptosis to promote metastatic colonization

**DOI:** 10.1186/s12935-024-03260-1

**Published:** 2024-02-18

**Authors:** Juan Sun, Jie Li, Kostas Pantopoulos, Yuqin Liu, Yixuan He, Weiming Kang, Xin Ye

**Affiliations:** 1 Department of General Surgery, Peking Union Medical College Hospital, Chinese Academy of Medical Sciences and Peking Union Medical College, No. 1 Shuaifuyuan, Dongcheng District, Beijing, 100730 China; 2 Lady Davis Institute for Medical Research, Jewish General Hospital, and Department of Medicine, McGill University, Montreal, QC Canada; 3 Department of Pathology, Institute of Basic Medical Sciences, Chinese Academy of Medical Sciences and Peking Union Medical College, Beijing, China

**Keywords:** Gastric cancer, Ferroptosis, Anoikis, Epithelial-mesenchymal transformation, Metastatic colonization

## Abstract

**Background and purpose:**

Ferroptosis is a form of regulated cell death characterized by iron-dependent lipid peroxidation. Its role in cancer metastasis remains unclear. In this study, we aimed to investigate the potential involvement of ferroptosis in gastric cancer (GC) metastasis.

**Methods:**

GC cells (AGS, MKN45, HGC27) were used to explore the role of ferroptosis in single and clustered cells with extracellular matrix (ECM) detachment in vitro. We overexpressed glutathione peroxidase 4 (GPX4) to inhibit ferroptosis and assessed the changes in cell proliferation, migration, invasion, and epithelial-mesenchymal transition (EMT). Then tumor tissues from 54 GC patients with and without lymphatic metastasis were collected for immunohistochemical staining to investigate the expression of ferroptosis and EMT markers. Finally, Kaplan–Meier survival curves were used to investigate the relationship between overall survival and expression of GPX4 in 178 GC patients.

**Results:**

Detached single cells had lower viability than adherent cells, but cell clustering improved their survival under matrix-detached conditions. Detached single cells exhibited an induction of iron-dependent reactive oxygen species (ROS) accumulation, glutathione (GSH) depletion, lipid peroxidation, upregulation of ACSL4, TFRC and HO-1, increased iron levels, and changes in mitochondrial morphology. Opposite effects were observed in detached clustered cells, including the upregulation of the ferroptosis suppressors GPX4 and SLC7A11. Overexpression of GPX4 inhibited ferroptosis and promoted GC cell proliferation, migration, invasion, and EMT. Immunohistochemical analysis of tumor tissues from GC patients indicated that lymphatic metastasis was associated with higher potential for ferroptosis inhibition and EMT induction. Finally, Kaplan–Meier survival curves demonstrated a significant decrease in overall survival among GC patients with high GPX4 expression.

**Conclusions:**

Our study provides the first evidence that inhibition of ferroptosis is a crucial mechanism promoting GC metastasis. GPX4 may be a valuable prognostic factor for GC patients. These findings suggest that targeting ferroptosis inhibition may be a promising strategy for GC patients with metastatic potential.

*Trial registration* The ethical approval code of this study in Institutional Review Board of Peking Union Medical College Hospital is No: K1447.

**Supplementary Information:**

The online version contains supplementary material available at 10.1186/s12935-024-03260-1.

## Introduction

Gastric cancer, with an estimated 1.27 million incident cases and 957,000 deaths in 2019 worldwide, is a leading cause of cancer incidence and mortality [1]. The incidence of GC is geographically distinct across the world, with a high prevalence in Asia, Africa, South America and eastern Europe [2]. It is the third most common malignancy and the third leading cause of cancer-related mortality worldwide in 2022 in China [3]. The 5-year survival rate is about 30%, while the 5-year survival rate of patients with metastatic GCs is less than 10% [4]. Among these GC patients, up to 65% present with stage III or IV disease in the US, while half of them present with advanced disease in Japan [5]. Notably, 20–60% of patients with locally advanced GC and no microscopically detectable residual cancer following surgical resection will eventually develop recurrence or metastasis [6]. Liver, lung, bone and peritoneum are all friendly sites for GC cells to grow and form metastatic lesions [7], which generally display remarkably low responsiveness to initial treatment and have a high risk of post-treatment relapse.

The development of metastasis including detachment of tumor cells from matrix (anoikis) [8], local invasion and migration, intravasation, circulation survival, extravasation and secondary site colonization [9]. Cancer cells must develop anoikis resistance to survive in circulation before forming metastatic foci in distant organs [10]. Anoikis could follow either the intrinsic pathway, due to the perturbation of mitochondria, or the extrinsic pathway triggered by cell surface death receptors [11]. In the intrinsic pathway, anoikis markedly stimulates the generation of reactive oxygen species (ROS) specifically in the mitochondria of detached cells and excess ROS would damage cellular macromolecular components and cause cell death [9]. Interestingly, ferroptosis—a form of regulated cell death that mainly relies on iron-mediated oxidative stress and subsequent cell membrane damage was first discovered by Dixon et al. in 2012, confirming the central role of ROS in ferroptotic cancer cell death execution [12]. Recent studies have also shown that anoikis can trigger ferroptosis in breast and prostate cancers [8, 13, 14]. However, whether anoikis triggers ferroptosis in GC and how GC cells resistant anoikis to survive to promote the metastatic colonization remains unclear.

In this study, we demonstrate that detached single GC cells undergo ferroptosis leading to the decreased survival and a reduction in metastatic capacity, while the clustering status of detached cells exhibit tolerance to ferroptosis compared with detached single cells. And then through overexpressing GSH peroxidase 4 (GPX4), a lipid repair enzyme protecting against oxidative damage and enabling cells to resist ferroptosis [15], we found that the inhibition of ferroptosis can significantly enhance the proliferation, migration and invasion of GC cells in vitro and in vivo. Our work reveals the role of ferroptosis in the metastasis of GC and understands the survival mechanisms in detached clustered cells, which are essential for the development of effective potential therapeutic strategy to inhibit the progression and metastasis of advanced GC.

## Materials and methods

### Cell culture and construction of matrix-detachment cells model

Human gastric cancer cell lines AGS, MKN45, HGC27, PUMC-HGC1 cell lines of human gastric cancer were bought from the Cell Resource Center of Peking Union Medical College (Beijing, China). All cells were cultured in RPMI 1640 medium, supplemented with 10% fetal bovine serum (Gibco, CA, USA) and 1% streptomycin/penicillin, and were cultured in a humidified atmosphere at 37 °C with 5% CO_2_. Cells grown in adherent conditions were cultured in normal cell-cultured plates while cells grown in detached clustered conditions were cultured in plates coated with poly 2-hydroxyethyl methacrylate (polyHEMA; 30 mg/ml; Beyotime, Shanghai, China) and dried overnight in laminar flow hood for the times noted [8]. To prevent cell clustering in detached cells, cells were treated with 2 mM EDTA [13] to get detached single ones.

### Measurement of cell viability

Cell viability was evaluated using the Cell Counting Kit-8 (CCK-8) (C6050, NCM Biotech, Suzhou, Jiangsu, China) according to the manufacturer’s instructions. Briefly, cells were trypsinized and complete medium was used to quench the trypsin. After counted, cells were seeded in adherent or detached wells of 96-well plates at a cell density of 1 × 10^4^ cells per well for appropriate times. Then after CCK-8 reagent was added to each well for 1 h incubation at 37 °C, the absorbance was measured on a microplate reader (Epoch, Bio-Tek, USA) at 450 nm.

### Malondialdehyde (MDA) assay

To assay lipid peroxidation, the Lipid Peroxidation MDA Assay Kit (S0131S, Beyotime, China) was used according to manufacturer’s specifications. Briefly, cells at a density of 1 × 10^6^ per well were cultured on six-well plates with and without poly-HEMA for 4 h. Then cells were collected, lysed with cell lysis solution (P0013, Beyotime, China) and reacted with thiobarbituric acid for 15 min at 100 °C. The absorbance was measured on a microplate reader (Epoch, Bio-Tek, USA) at 532 nm and MDA level was normalized to protein concentration which was assayed using a Beyotime BCA Protein Assay Kit (P0010).

### GSH assay

The measurement of glutathione was using a Reduced glutathione assay kit (#A006-2-1, Jiancheng, Nanjing, China) according to manufacturer’s specifications. And the absorbance was measured on a microplate reader (Epoch, Bio-Tek, USA) at 420 nm and GSH level was normalized to protein concentration.

### Measurement of ROS

The Reactive Oxygen Species Assay Kit (S0033S, Beyotime, China) was used to detect intracellular ROS. Cells were seeded into six-well plates (plates for detached group were coated with poly-HEMA one day before) and collected to tubes after incubated for the appropriate time, washed twice with serum-free medium and incubated 30 min with DCFH-DA at a final concentration of 10 μM in serum-free medium at 37 °C, then washed three times with serum-free medium. The level of ROS was determined by flow cytometer (LSRFortessa, BD, USA).

### FerroOrange iron assay

Fluorescent indicator FerroOrange (F374; Dojindo Laboratories Inc.) was used to detect the intracellular iron. Briefly, 1 × 10^6^ cells per well were seeded on six-well plates and cultured for 4 h, collected and incubated with 1 µmol/L FerroOrange working solution for 30 min. The stained cells were passed through a cell strainer and photographed by laser scanning confocal microscope (Nikon). The relative mean fluorescence intensity of intracellular ions was calculated and analyzed by ImageJ.

### Western blot analysis

Cells were lysed in the RIPA buffer (Beyotime, China) with Halt Protease and Phosphatase inhibitor Cocktail (Thermo Scientific, Rockford, IL, USA). After centrifuging at 14,000 rpm for 15 min at 4 °C, cell lysates were determined protein concentration using BCA Protein Assay Kit and separated via 10% SDS-PAGE (Beyotime, China) for nitrocellulose membrane blotting. Blocked with 5% skim milk for 1 h, the blotted membranes were incubated with the primary antibody overnight at 4 °C. Then immunoreactive bands were incubated with corresponding secondary antibody for 1 h at room temperature and visualized by ECL Plus (NCM Biotech, Suzhou, Jiangsu, China) using a SuperSignal West Pico PLUS Chemiluminescent Substrate (Thermo Scientific, Rockford, IL, USA). The antibodies against GPX4 (ab125066) and SLC7A11 (ab175186) were purchased from Abcam (Cambridge, MA, USA). Antibodies against TFRC (10084-2-AP), HO-1 (66743-1-lg), ACSL4 (66617-1-lg), E-cadherin (20874-1-AP), ZO-1 (66542-1-lg), N-cadherin (22018-1-AP), Vimentin (10366-1-AP), and β-actin (66009-1-lg) were purchased from Proteintech (Wuhan, China).

### Mito-tracker green staining

The MitoTracker Green (#C1048, Beyotime, China) and Hoechst 33342 (#C1028, Beyotime, China) were used to visualize mitochondria and nuclei. In brief, Cells were seeded in prepared six-well plates for 4 h, collected to tubes, incubated with 50 nM MitoTracker Green for 20 min and then added 10 μM Hoechst to co-cultured for 10 min. After washing three times with PBS, cells were photographed by laser scanning confocal microscope (Nikon) and the relative mean fluorescence intensity of mitochondria was calculated and analyzed by ImageJ.

### Transmission electron microscopy (TEM)

Cells were collected, centrifuged and fixed in 2.5% glutaraldehyde (EM Grade, P1126; Solarbio Life Sciences, Beijing, China) at 4 °C overnight. Then, after fixing in 1% osmium acid and dehydrating, the samples were embedded in molds. Ultrathin section (0.08 µm) were stained with lead citrate and uranyl acetate, and then analyzed by TEM (JEM-1400Plus; JEOL Ltd., Tokyo, Japan).

### Overexpressing GPX4

A lentiviral transfection was established to overexpress GPX4 and the lentiviral vectors packaged with full-length GPX4 were bought from GENE (Shanghai, China). AGS and MKN45 were transfected with GPX4 overexpression lentivirus or its negative control based on the relative expression level of GPX4 in four gastric cancer cell lines. Then screening with puromycin (Beyotime, China) for 2 weeks to obtain stable cell lines and verifying by Western blotting.

### Colony formation assay

A total of 1 × 10^3^ cells / well were seeded into six-well plates and incubated for two weeks. The visible colonies were fixed with 4% paraformaldehyde, stained with 0.1% crystal violet and then imaged and counted.

### Transwell and wound healing

For transwell invasion, a total of 2 × 10^4^ /ml cells were suspended in 200 µL of serum-free medium and added into upper chamber of 24-well transwell plate (8 μM pore size; Corning), which was coated with Matrigel (Corning, 354234), and 600 µL of medium containing 20% FBS was added into the lower chamber. After incubation for the appropriate time, cells on the lower surface of membrane were fixed in 4% paraformaldehyde, stained by 0.1% crystal violet and then counted under the light microscope (Nikon). For transwell migration, the upper chamber was not coated with Matrigel.

For wound healing assay, a total of 5 × 10^5^ /ml cells were seeded into six-well plates and scratched with a sterile plastic tip when they reached almost 100% confluency, then cultured with serum-free medium after washing with PBS twice. Photographs of random fields across three replicate wells were captured for analysis under light microscope (Nikon).

### Collection of clinical samples

A total of 54 tumor tissues (25 with lymphatic metastasis while 29 without) were obtained from GC patients who accepted radical gastrectomy and pathologically diagnosed as gastric adenocarcinoma at the Department of General Surgery, Peking Union Medical College Hospital, China, in 2021–2022. All specimens were collected within 5 min after resection and immediately transferred to a − 80 °C refrigerator or embedded in paraffin. We also analyzed the correlation between GPX4 expression levels (through IHC) in 178 tumor tissues and the overall survival time of corresponding GC patients who underwent radical gastrectomy at our hospital from 2015 to 2017 and were pathologically diagnosed with gastric adenocarcinoma. This study was reviewed and approved by the Institutional Review Board of Peking Union Medical College Hospital. Conducted in accordance with the guidelines set by the Declaration of Helsinki, the written informed consent of each patient or their relatives was obtained for the collection of specimens.

### Immunohistochemistry (IHC)

The paraffin-embedded tumor tissues were sectioned (thickness, 4 µm) and deparaffinized in xylene, rehydrated through graded alcohol. Then they were treated with 3% H_2_O_2_ for 10 min at room temperature to block endogenous peroxidase activity. Next, the sections stained using streptavidin peroxidase-conjugated method with GPX4, SCL7A11, TFRC, ACSL4, N-cad, E-cad, Vimentin and ZO-1 as their specific primary antibodies. Field emission scanning electron microscope (Janpan, SU8010) was utilized to capture images of the tissue, while Image-Pro Plus software was used for calculating the expression level of proteins.

### Statistical analysis

All results were shown as mean ± standard deviation. Graphpad Prism 8.0 (CA, USA) and SPSS 27.0 were used for the analysis, and P-value < 0.05 was considered statistically significant.

## Results

### Clustering of matrix-detached cells rescues anoikis-induced cell death

**Fig. 1:**
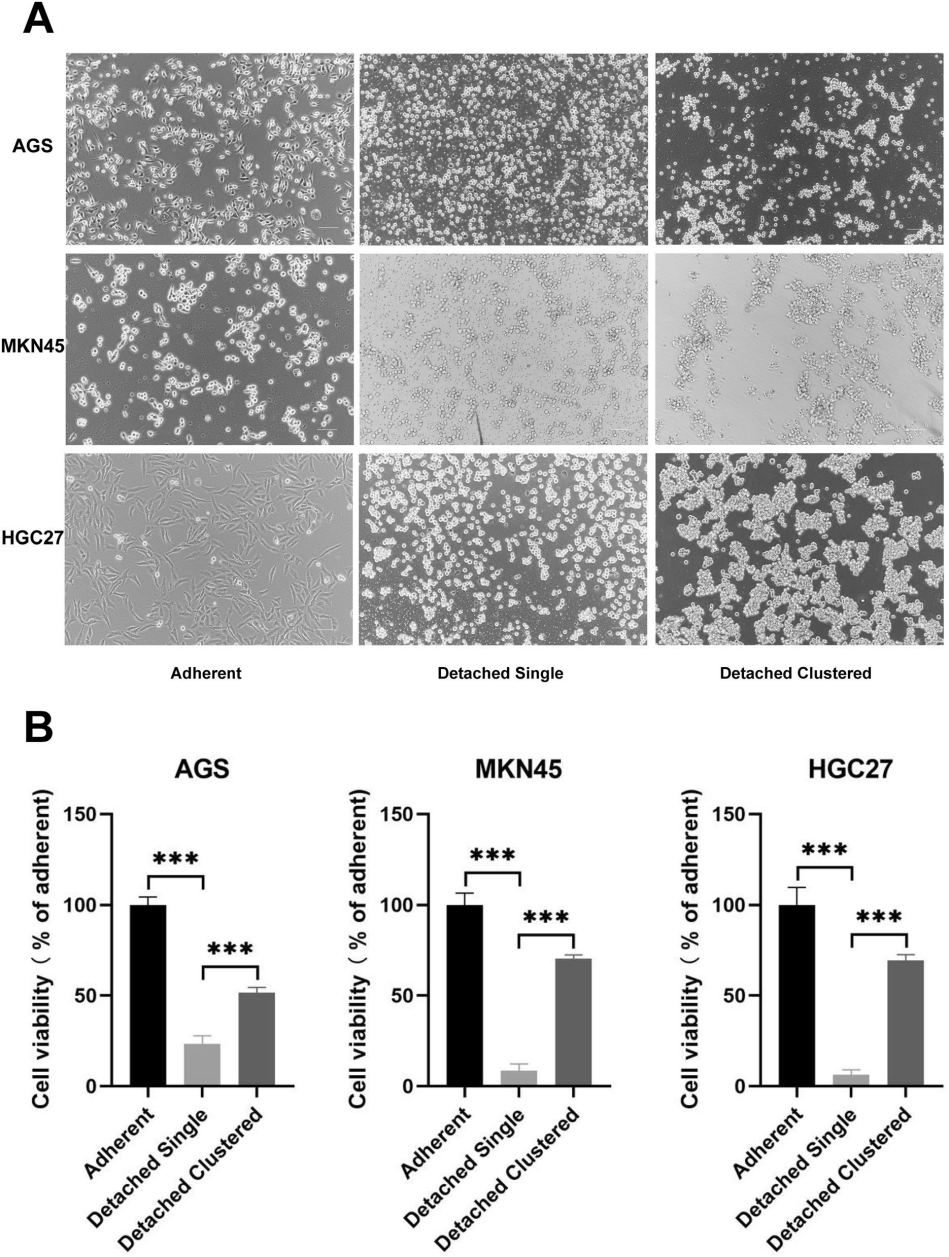
Clustering of matrix-detached cells rescues anoikis-induced cell death. **A** AGS, MKN45, HGC27 cells in attached (adherent) and detached status (detached clustered—allowed cells to cluster spontaneously; detached single—treated with either 2 mM EDTA). Scale bar = 100 µm. **B** The effect of detached single and detached clustered on the cell viability of GC cells (***P < 0.001)

Matrix-detached GC cells clustered spontaneously upon detachment and theses clusters could be dissociated into single cells by culturing in the presence of 2 mM EDTA [13, 16] (Fig. 1A). We observed that compared with adherent cells, detached cells that had been prevented from clustering showed a strong decrease in viability 24 h after matrix detachment. However, the clustering status could rescue their viability under matrix-detached conditions compared with detached single cells (Fig. 1B).

### Clustering of matrix-detached cells inhibits anoikis-induced ferroptosis

**Fig. 2:**
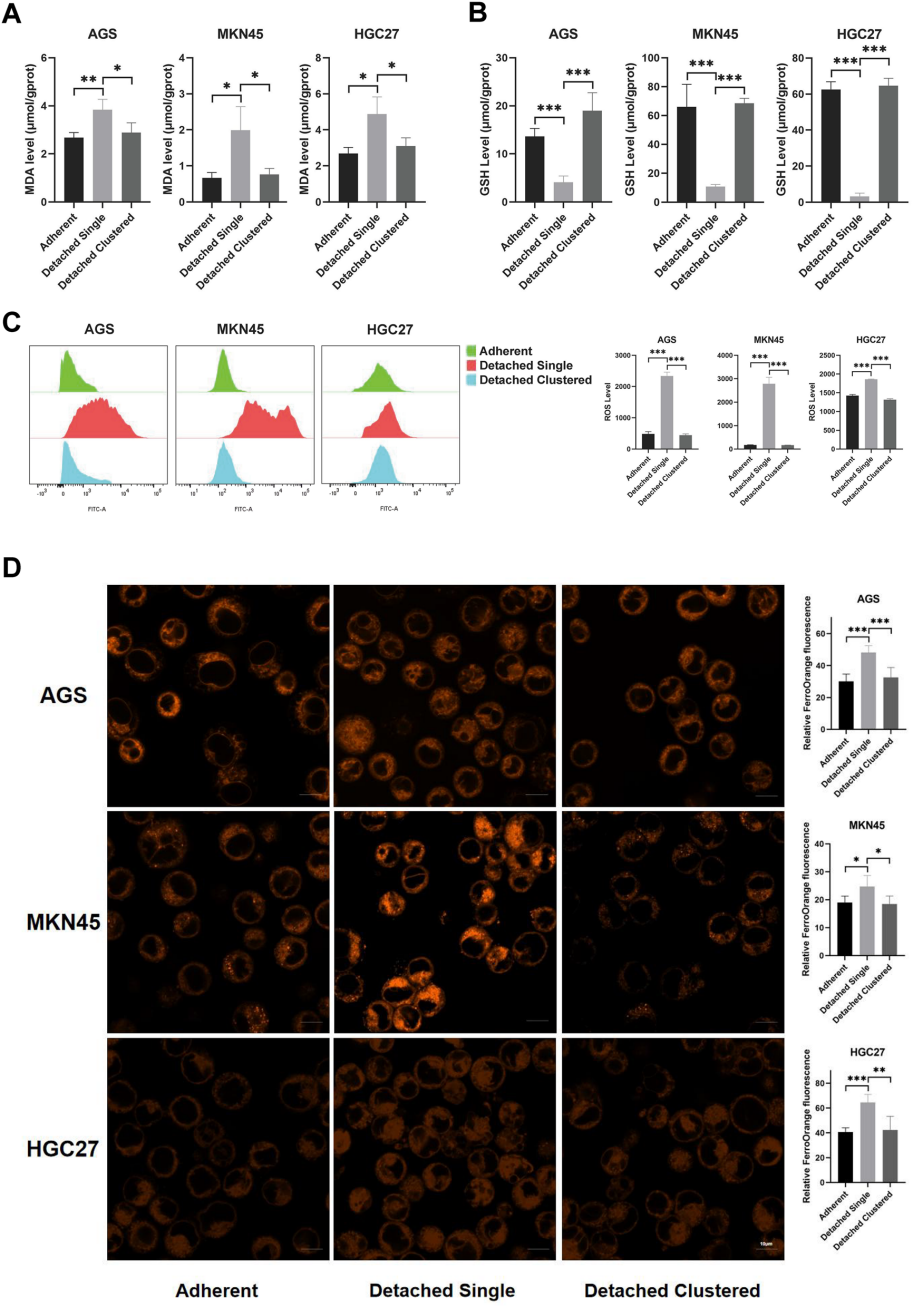
Impact of detached single and detached clustered status on ferroptotic events in AGS, MKN45 and HGC27. **A** Intracellular MDA levels of GC cells in adherent, detached single and detached clustered status. **B** Intracellular GSH levels of GC cells in three different statuses. **C** The cellular ROS level of GC cells in three different statuses were analyzed by a flow cytometer. **D** The iron ion level and their qualifications of GC cells in three different statuses were determined using the fluorescent indicator FerroOrange. Scale bar = 10 µm. (*P < 0.05, **P < 0.01, ***P < 0.001)

To explore whether ferroptosis was involved in the death of detached single cells and the inhibition of death of detached clustered cells, we analyzed several ferroptotic markers. As expected, the oxidative stress marker MDA also showed a significant increase in detached single AGS, MKN45 and HGC27 cells when compared with adherent cells, as well as a significant decrease in detached clustered cells when compared with detached single cells (Fig. 2A). By contrast, GSH levels were remarkably decreased in detached single cells, indicating GSH depletion in this group (Fig. 2B). Besides, ROS accumulation was significantly enhanced in detached single cells compared to adherent and detached clustered cells (Fig. 2C).

**Fig. 3:**
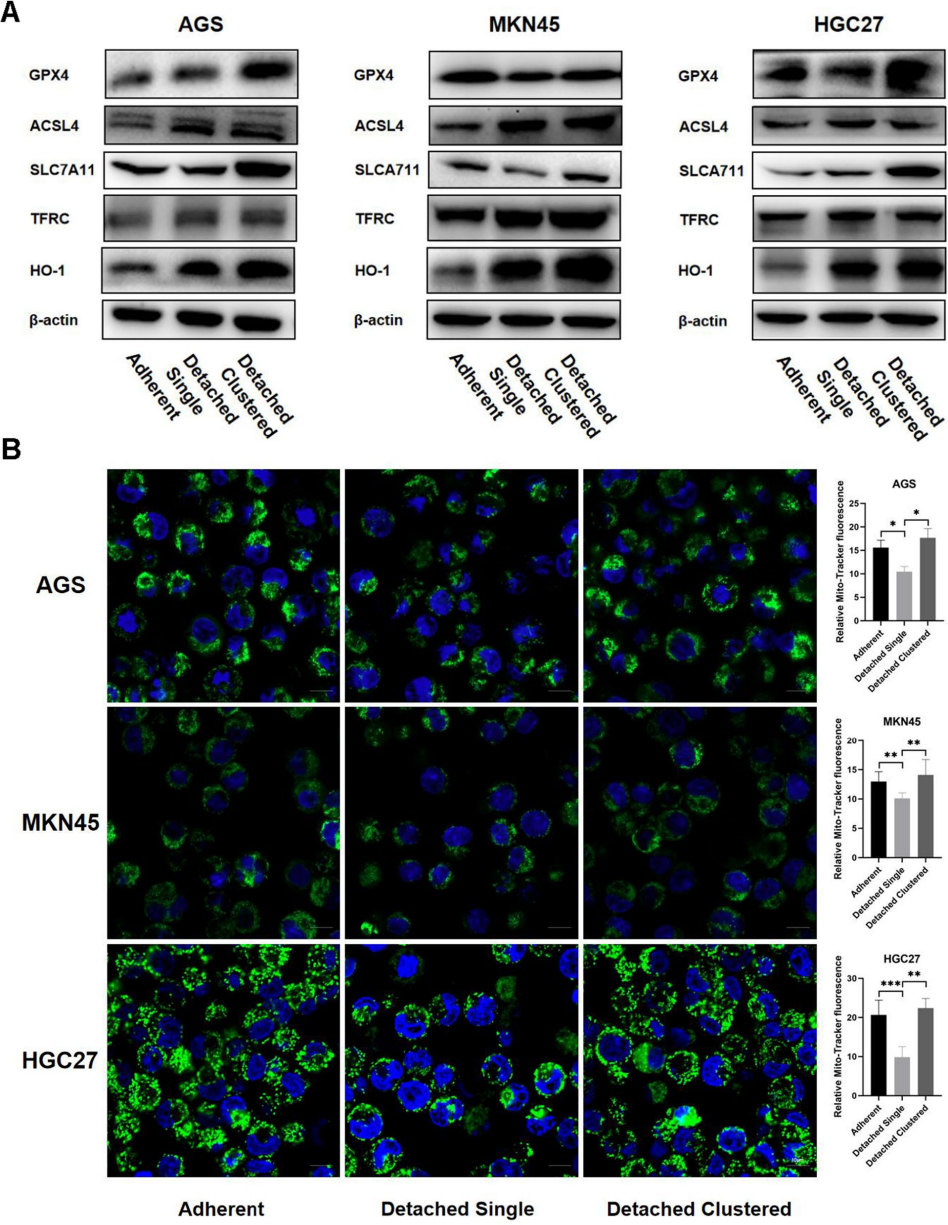
Impact of detached single and detached clustered status on expression of ferroptosis-related proteins and mitochondrial staining. **A** The expression of positive regulatory proteins for ferroptosis (ACSL4 and TFRC) and the negative regulatory proteins for ferroptosis (GPX4 and SLC7A11) were detected by western blotting of GC cells in three different statuses. **B** The Mitochondria morphology was assessed with Mito-Tracker Green and qualified with Image J. Scale bar = 10 µm. (*P < 0.05, **P < 0.01, ***P < 0.001)

**Fig. 4:**
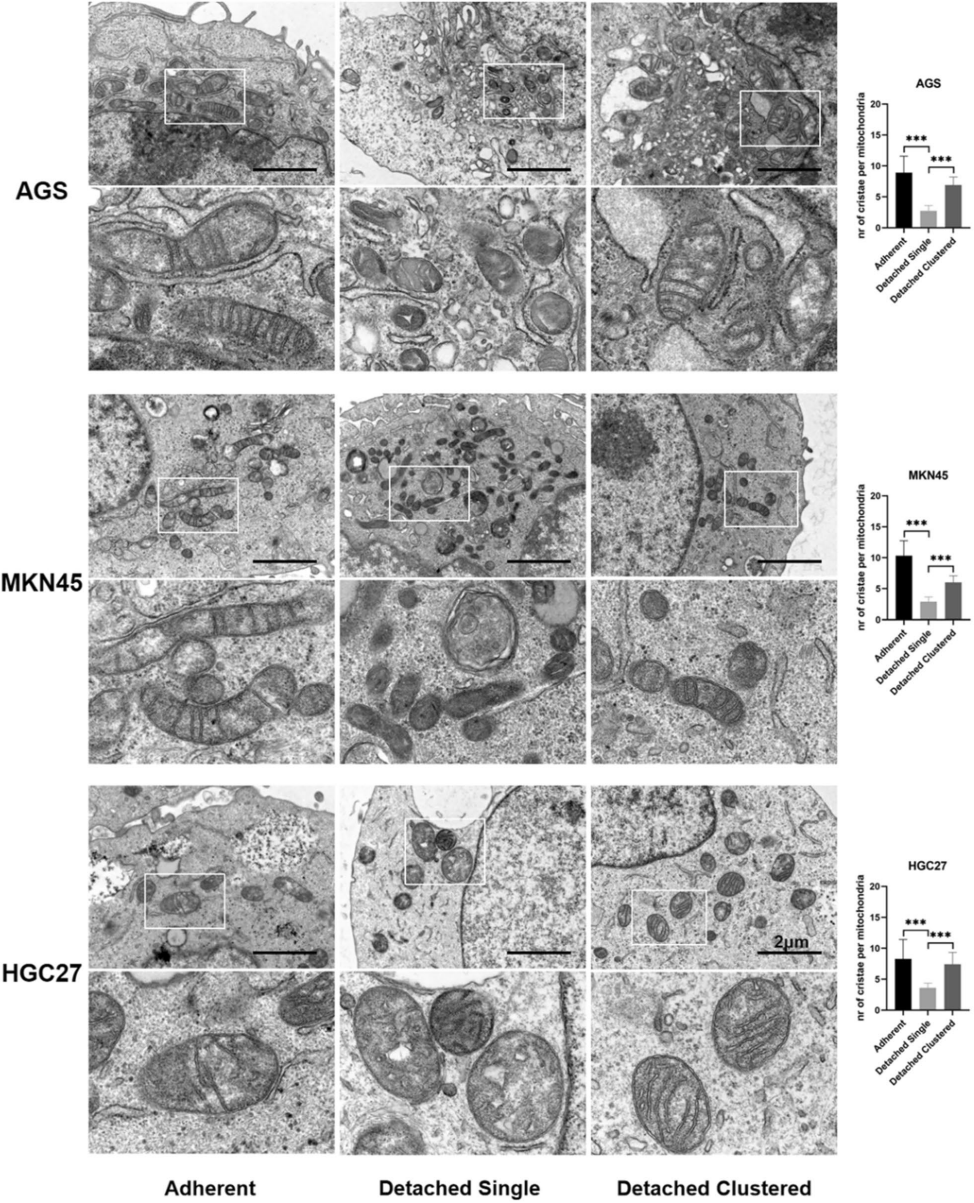
Representative transmission electron microscopy images of mitochondria of adherent and detached cells. Scale bars represent 2 µm. Graphs present quantified number of cristae per mitochondria. Data are presented as mean ± SD. ***P < 0.001

It is well known that iron is an essential stimulus for ferroptosis. Therefore, we used the fluorescent indicator FerroOrange to examine accumulation of intracellular redox active iron in GC cells. As showed in Fig. 2D, compared with adherent and detached clustered cells, an increase of the proportion of FerroOrange-positive cells was found in detached single cells. Along with the increased iron levels, Western blotting was performed to monitor the expression of ferroptosis-related proteins. We found that expression of the ferroptosis inducers ACSL4, TFRC and HO-1 was significantly increased in detached single cells as compared to adherent control cells, while expression of the ferroptosis-suppressors GPX4 and SLC7A11 was significantly increased in detached cluster vs single cells (Fig. 3A and Additional file 1: Figure S1). Changes of mitochondrial morphology are another phenotypic hallmark of ferroptosis. Thus, probing with Mito-Tracker Green showed a significant decrease of mitochondrial fluorescence in detached single cells (Fig. 3B). Moreover, transmission electron microscopy revealed a striking alteration in mitochondrial morphology, with significantly fewer cristae per mitochondrion in detached single cells (Fig. 4).

Taken together, these findings strongly suggest that detached single cells undergo ferroptotic cell death, while the clustering of detached cells can inhibit anoikis-induced ferroptosis to maintain cell survival in the circulation.

### Inhibition of ferroptosis promotes proliferation, migration, invasion and EMT of GC cells

**Fig. 5:**
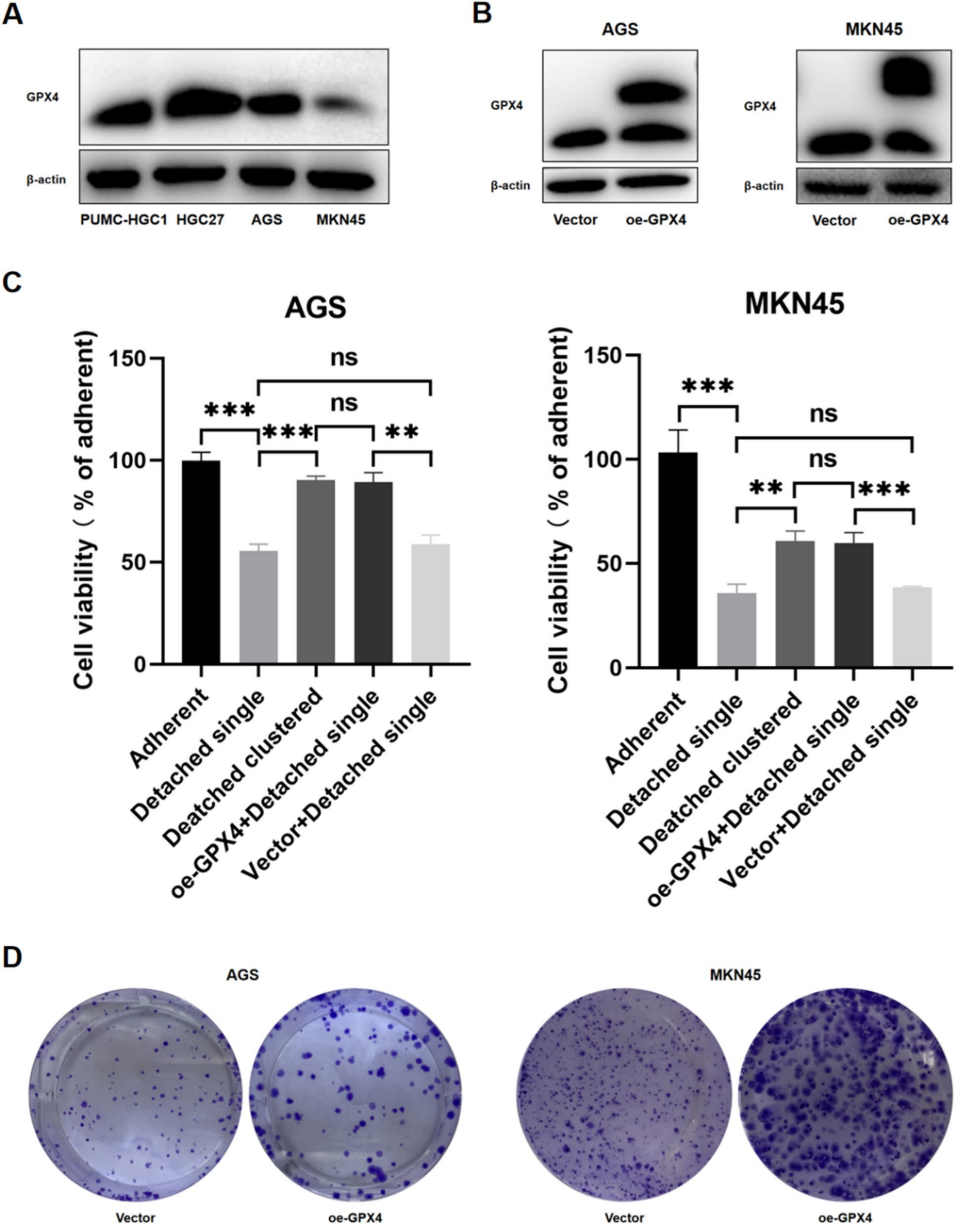
Impact of inhibiting ferroptosis on viability and proliferation of AGS and MKN45 cells. **A** The baseline expression level of GPX4 in four gastric cancer cell lines. **B** The overexpression of GPX4 in AGS and MKN45. **C** Overexpressing GPX4 rescued viability of detached single AGS and MKN45 cells. (**P < 0.01, ***P < 0.001). **D** Cell colony formation assays of the two GPX4-overexpressed cell lines

**Fig. 6:**
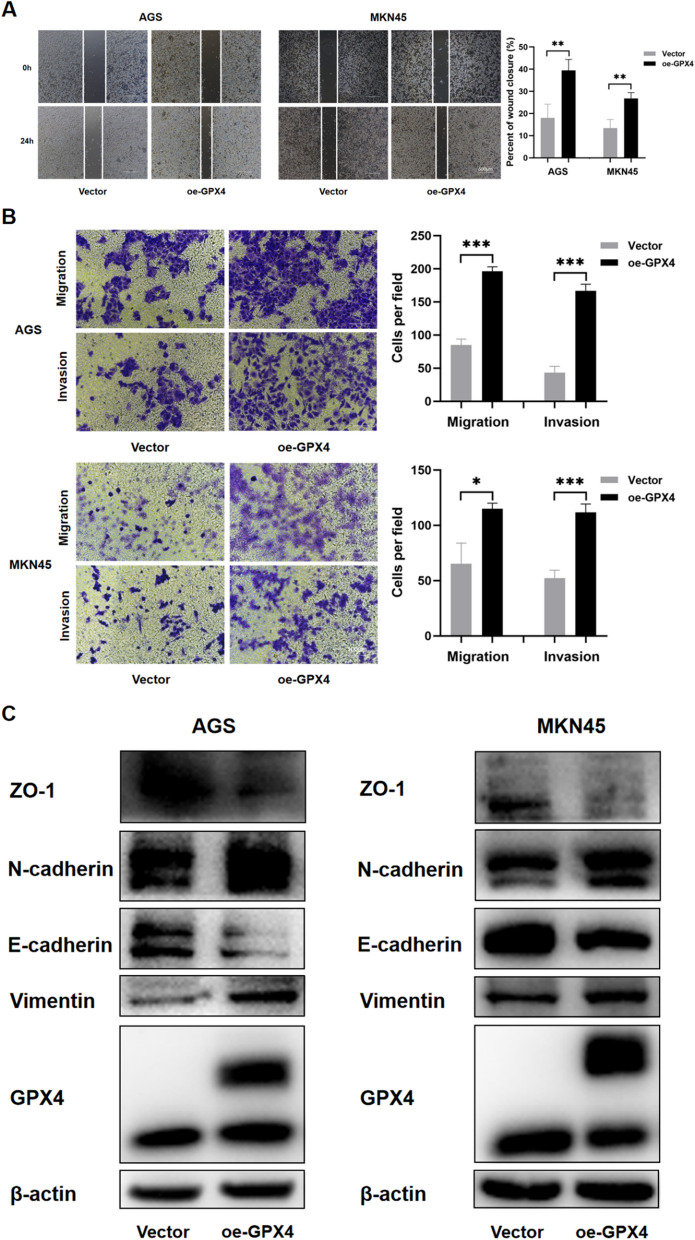
Ferroptosis inhibition promotes migration, invasion and EMT of AGS and MKN45 cells. **A** Representative results of wound healing assay in GPX4-overexpressed AGS and MKN45 cells after 24 h of incubation. The images show the extent of wound closure in the cells. Scale bar = 500 µm. The mean ± sd. is shown. **P < 0.01. **B** Migration and invasion assays were performed by the 24-transwell system. Scale bar = 500 µm. The mean ± sd. is shown. *P < 0.05, ***P < 0.001. **C** The expression of several key EMT markers Vimentin, E-Cadherin, N-Cadherin and ZO-1 were detected by western blotting of GPX4-overexpressed AGS and MKN45 cells

To investigate whether the inhibition of ferroptosis affected cell proliferation, migration and invasion, we compared 4 GC cell lines for GPX4 expression (Fig. 5A) and chose two of them with the lowest GPX4 levels (AGS and MKN45) for GPX4 overexpression (Fig. 5B). We specifically investigated the levels of MDA, GSH, and ROS in detached single state GPX4-overexpressing and vector control cells. The results revealed that in the detached single status, GPX4-overexpressing cells displayed lower levels of MDA and ROS, and higher levels of GSH compared to vector cells. This indicates that GPX4 overexpression can inhibit ferroptosis induced by the detached single state (Additional file 2: Figure S2). Then we verified that GPX4 overexpression could rescue cell viability of detached single cells (Fig. 5C). Cell colony formation assays also demonstrated that inhibiting ferroptosis promoted the proliferation of GC cells. Thus, colonies of oe-GPX4 cells were bigger than those from vector-transfected controls, although colony numbers did not differ among the two groups (Fig. 5D). Wound healing and transwell assays showed that GPX4 overexpression led to increased migration and invasion (Fig. 6A, B). Next, we analyzed the expression of several EMT-associated markers by Western blotting. As shown in Fig. 6C, GPX4 overexpression promoted down-regulation of the epithelial marker E-cadherin and ZO-1, and up-regulation of the mesenchymal markers Vimentin and N-cadherin. Collectively, these results suggest that inhibition of ferroptosis promotes EMT in GC cells.

### Ferroptosis and EMT in GC patients with and without lymphatic metastasis

**Fig. 7:**
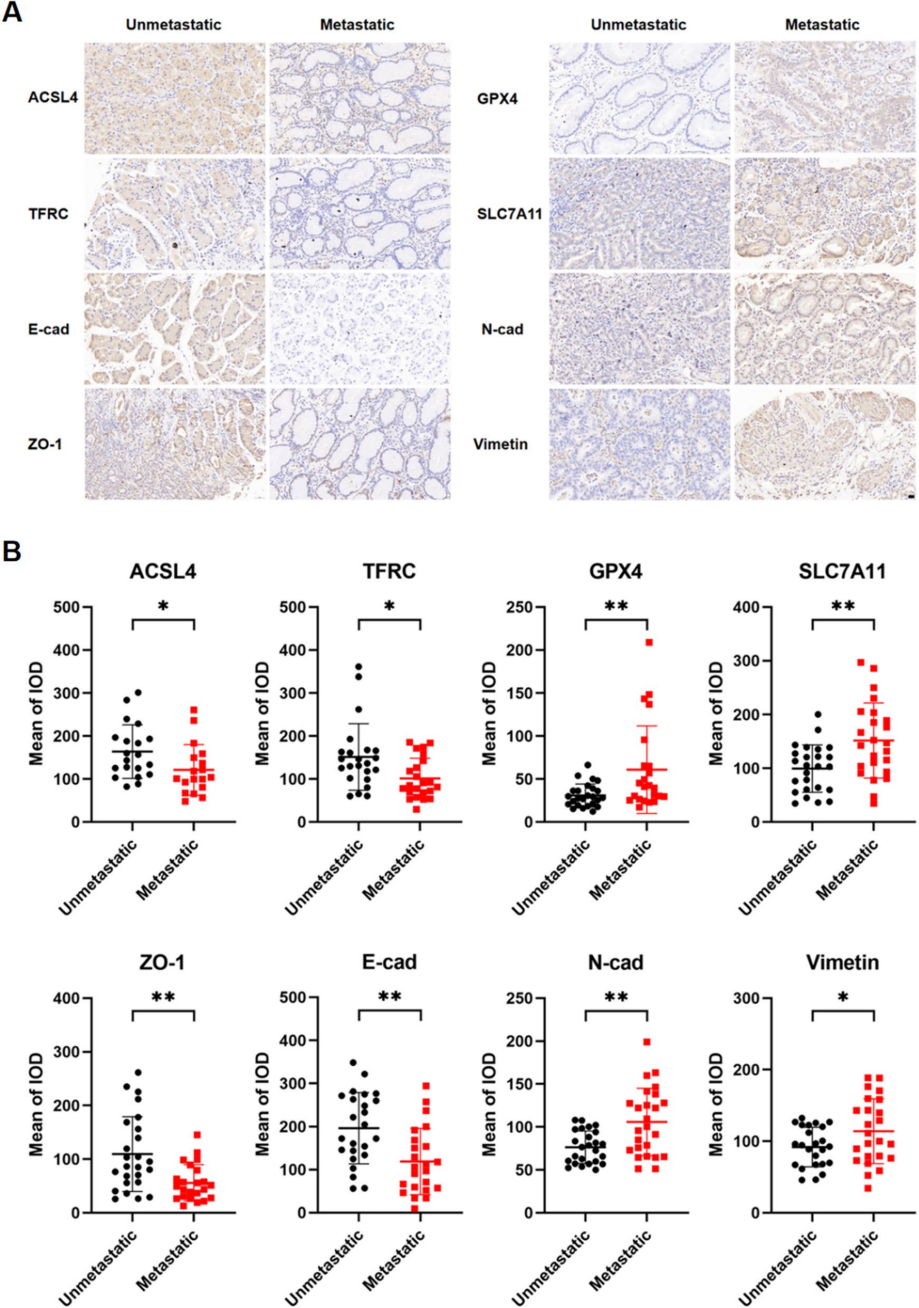
Ferroptosis and EMT markers in GC patients with and without lymphatic metastasis. **A** Representative immunohistochemical staining (400X) of ferroptosis and EMT markers in tumor tissues from gastric cancer (GC) patients with and without lymphatic metastasis. Scale bar = 20 µm. **B** IHC staining quantification by mean of integrated optical density (IOD) using Image-Pro Plus in different markers and groups. (*P < 0.05, **P < 0.01)

The clinical and pathological characteristics of 54 GC patients are described in Table 1. It is noteworthy that patients with lymphatic metastasis exhibited significantly larger tumor sizes, poorer differentiation, and higher T stages when compared to those without metastasis. Next, we performed immunohistochemical staining to investigate the expression of regulatory proteins associated with ferroptosis (GPX4, TFRC, SLC7A11, and ACSL4) and EMT (E-cad, N-cad, Vimentin, and ZO-1). As shown in Fig. 7A and B, we found high expression of GPX4 and SLC7A11 and low expression of ACSL4 and TFRC in lymphatic metastatic samples, indicating inhibition of ferroptosis in these tumors. In addition, lymphatic metastasis was associated with increased expression of N-cad and Vimentin, and reduced levels of E-cad and ZO-1. Taken together, our results suggest that the inhibition of ferroptosis and the induction of EMT play a crucial role in the metastatic progression of GC.

**Table 1:** The clinical and pathological characteristics of 54 patients with and without lymphatic metastasis

	Unmetastatic	Metastatic	P value
No	29	25	–
Age (years)	63.93 ± 6.92	59.40 ± 10.72	0.067
BMI (kg/m^2^)	24.35 ± 3.22	23.13 ± 4.25	0.830
Inpatient days	25.41 ± 4.656	16.08 ± 6.22	0.655
Tumor size (cm)	2.39 ± 1.04	4.19 ± 2.83	0.002^**^
Sex			
Males	18 (62.07%)	17 (68.00%)	0.649
Females	11 (37.93%)	8 (32.00%)	
Tumor location			
Fundus	4 (14.29%)	6 (24.00%)	0.320
Body	12 (42.86%)	13 (52.00%)	
Antrum	12 (42.86%)	6 (24.00%)	
Differentiation			
Well	2 (6.90%)	1 (4.00%)	0.047*
Moderate	10 (34.48%)	2 (8.00%)	
Moderate to Poor	10 (34.48%)	8 (32.00%)	
Poor	7 (24.14%)	14 (56.00%)	
Lauren type			
Intestinal-type	12 (52.17%)	6 (26.09%)	0.102
Diffuse	4 (17.40%)	10 (43.48%)	
Mixed-type	7 (30.43%)	7 (30.43%)	
T stage			
1	16 (55.17%)	0 (0.00%)	< 0.001***
2	8 (27.59%)	13 (52.00%)	
3	3 (10.34%)	8 (32.00%)	
4	2 (6.90%)	4 (16.00%)	
N stage			
0	29 (100%)	0 (0.00%)	< 0.001***
1	0 (0.00%)	9 (36.00%)	
2	0 (0.00%)	5 (20.00%)	
3	0 (0.00%)	11 (44.00%)	
M stage			
0	29 (100%)	25 (100%)	–
1	0 (0.00%)	0 (0.00%)	

Statistical significance was determined by t-test and Chi-square test. (*P < 0.05, **P < 0.01, ***P < 0.001)

### High expression of GPX4 in GC tissue correlates with poor prognosis of patients

**Fig. 8:**
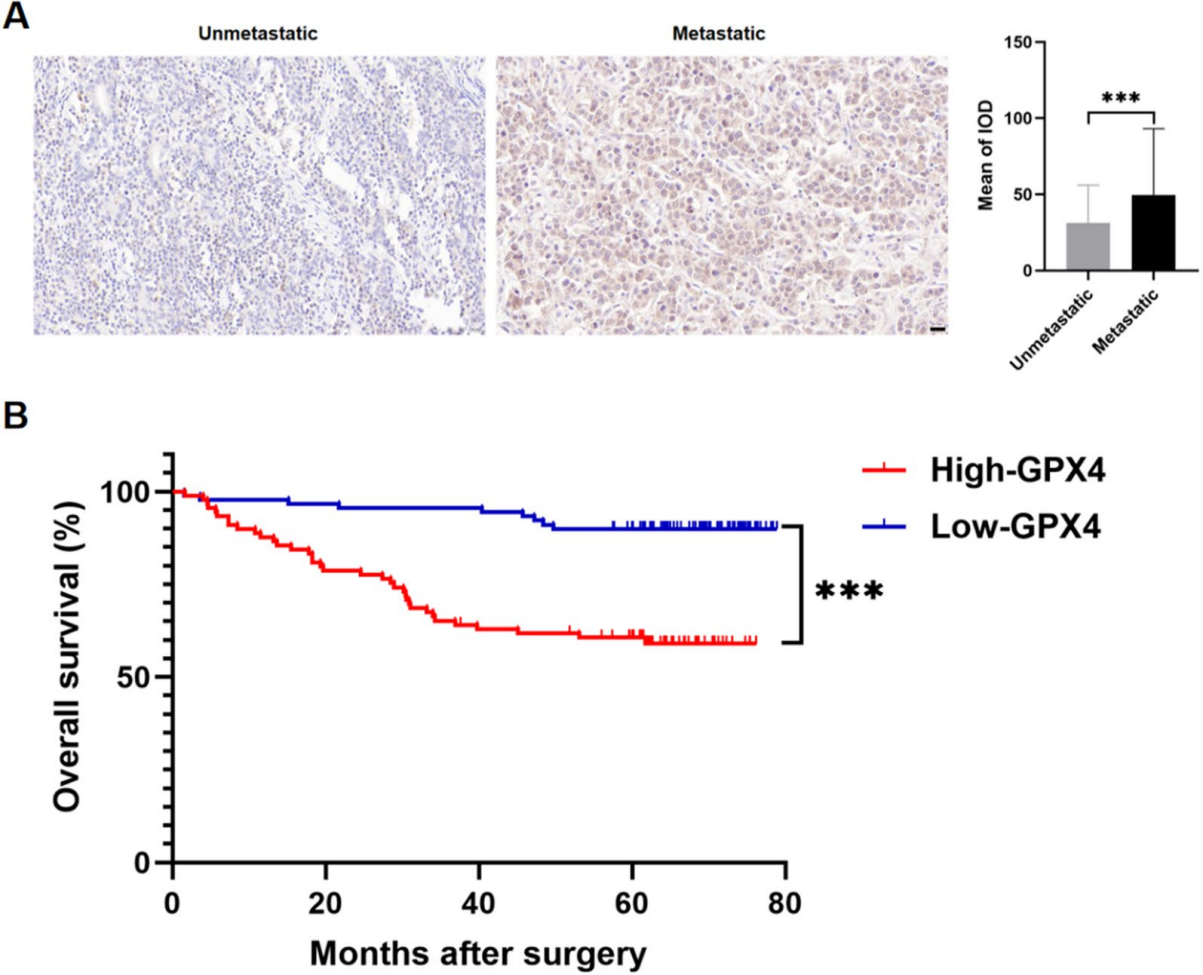
The prognostic value of GPX4 in patients with GC. **A** Representative immunohistochemical staining (400X) of GPX4 in tumor tissues from gastric cancer (GC) patients with and without lymphatic metastasis (Scale bar = 20 µm). Quantification of IHC staining is shown (***P < 0.001). **B** Comparison of overall survival (OS) between patients with high expression of GPX4 and low expressing cases in GC via Kaplan–Meier analysis. (***P < 0.001)

Table 2 provided the analysis of clinical and pathological characteristics of 178 GC patients, whose overall survival (OS) time was completed, grouped by lymphatic metastasis status. There were no significant differences in age, BMI, sex, tumor location, Lauren type, or M stage between the two groups. However, patients with lymphatic metastasis exhibited poorer differentiation, higher TNM stages, and more utilization of adjuvant chemotherapy when compared to those without metastasis. Then, we performed immunohistochemical staining to investigate the expression of GPX4. As shown in Fig. 8A, we found high expression of GPX4 in lymphatic metastatic samples, which further validated the results of Fig. 7.

**Table 2:** The clinical and pathological characteristics of 178 patients with and without lymphatic metastasis

	Unmetastatic	Metastatic	P value
No	93	85	–
Age (years)	59.35 ± 10.21	60.20 ± 11.57	0.605
BMI (kg/m^2^)	23.99 ± 3.24	23.05 ± 3.66	0.071
Sex			
Males	69 (74.19%)	58 (68.24%)	0.380
Females	24 (25.81%)	27 (31.76%)	
Tumor location			
Fundus	14 (15.05%)	11 (12.94%)	0.853
Body	28 (30.11%)	24 (28.24%)	
Antrum	51 (54.84%)	50 (58.82%)	
Differentiation			
Well and Moderate	37 (39.78%)	18 (21.18%)	0.007**
Poor	56 (60.22%)	67 (78.82%)	
Lauren type			
Intestinal-type	38 (41.30%)	29 (34.12%)	0.466
Diffuse	28 (30.43%)	33 (38.82%)	
Mixed-type	26 (28.26%)	23 (27.06%)	
T stage			
1	63 (67.74%)	12 (14.12%)	< 0.001***
2	15 (16.13%)	16 (18.82%)	
3	10 (10.75%)	26 (30.59%)	
4	5 (5.38%)	31 (36.47%)	
N stage			
0	93 (100.00%)	0 (0.00%)	< 0.001***
1	0 (0.00%)	25(29.41%)	
2	0 (0.00%)	23(27.06%)	
3	0 (0.00%)	37(43.53%)	
M stage			
0	93 (100.00%)	84 (98.82%)	0.294
1	0 (0.00%)	1 (1.18%)	
TNM stage			
I	78 (83.87%)	6 (7.06%)	< 0.001***
II	14 (15.05%)	27 (31.76%)	
III	1 (1.08%)	51 (60.00%)	
IV	0 (0.00%)	1 (1.18%)	
Adjuvant chemotherapy			
Yes	26 (30.58%)	63 (74.12%)	< 0.001***
No	67 (78.82%)	22 (25.88%)	

Statistical significance was determined by t-test and Chi-square test. (**P < 0.01, ***P < 0.001)

Moreover, we further analyzed the clinical and pathological characteristics of these 178 GC patients based on the expression of GPX4 (Table 3). There were no statistically significant differences in these characteristics between patients with high and low expression of GPX4. However, the Kaplan–Meier survival curves demonstrated a significant decrease in the OS of GC patients with high GPX4 expression (Fig. 8B), suggesting that GPX4 could be a crucial prognostic factor for GC patients, as high GPX4 expression levels significantly correlate with reduced OS rates.

**Table 3:** Correlation between GPX4 expression and clinical features

	High-GPX4	Low-GPX4	P value
No	89	89	
Age (years)	60.87 ± 10.53	58.65 ± 11.12	0.174
BMI (kg/m^2^)	23.30 ± 2.88	23.78 ± 3.98	0.356
Sex			
Males	60 (67.4%)	67 (71.3%)	0.246
Females	29 (32.6%)	22 (24.7%)	
Tumor location			
Fundus	11 (12.4%)	14 (15.7%)	0.396
Body	23 (25.8%)	29 (32.6%)	
Antrum	55 (61.8%)	46 (51.7%)	
Differentiation			
Well and Moderate	28 (31.5%)	27 (30.3%)	0.871
Poor	61 (68.5%)	62 (67.9%)	
Lauren type			
Intestinal-type	35 (39.3%)	32 (36.4%)	0.463
Diffuse	33 (37.1%)	28 (31.8%)	
Mixed-type	21 (23.6%)	28 (31.8%)	
T stage			
1	32 (36.0%)	43 (48.3%)	0.188
2	20 (22.5%)	11 (12.4%)	
3	20 (22.5%)	16 (18.0%)	
4	17 (19.1%)	19 (21.3%)	
N stage			
0	48 (53.9%)	45 (50.6%)	0.836
1	13 (14.6%)	12 (13.5%)	
2	12 (13.5%)	11 (12.4%)	
3	16 (18.0%)	21 (23.6%)	
M stage			
0	88 (98.9%)	89 (100%)	0.316
1	1 (1.1%)	0 (0%)	
TNM stage			
I	40 (44.9%)	44 (49.4%)	0.277
II	25 (28.1%)	16 (18.0%)	
III	23 (25.8%)	29 (32.6%)	
IV	1 (1.1%)	0 (0%)	
Adjuvant chemotherapy			
Yes	46 (51.7%)	43 (48.3%)	0.653
No	43 (48.3%)	46 (51.7%)	

Statistical significance was determined by t-test and Chi-square test

## Discussion

Our study investigates the role of ferroptosis in gastric cancer metastasis. Detached single GC cells undergo ferroptotic cell death, while clustered cells exhibit tolerance to ferroptosis. Overexpressing the ferroptosis suppressor GPX4 promotes GC cell proliferation, migration, invasion, and EMT. High GPX4 expression correlates with reduced overall survival in GC patients. Targeting ferroptosis inhibition may be a promising strategy for GC patients with metastatic potential.

To investigate mechanisms in the cell death of single cells and the rescue of clustered cells under matrix detachment, we examined the potential role of ROS. Labuschagne et al. [16] showed that detached cells that had been prevented from clustering showed a strong increase in mitochondrial ROS and a clear increase in oxidative stress compared to clustered cells. They also tested the effect of ouabain (an FDA-approved drug that can prevent the clustering of cancer cells) to rule out the influence of EDTA on the activity of several Mg^2+^-dependent metabolic enzymes; the results mirrored those seen following EDTA treatment. Interestingly, it was also shown that ROS were the actual executioners of death in cancer cells undergoing ferroptosis [12]. Our research findings indicate that when single cells become detached, they are more prone to undergoing ferroptotic cell death. However, when these detached cells cluster together, this collective organization can prevent anoikis-induced ferroptosis, thereby promoting the survival of cells within the circulation.

The ability of tumor cells to migrate, invade, and metastasize is largely attributed to the crucial role of epithelial-mesenchymal transition (EMT) [17]. Previous work showed that the EMT of therapy-resistant cancer cells could be inhibited by ferroptosis inducers [18]. We demonstrated that the clustering status of detached cells could inhibit anoikis-induced ferroptosis to promote cell survival, but whether ferroptosis is involved in other steps of GC metastasis remains unclear. Then, we selected AGS and MKN45 GC cell lines with low GPX4 expression and overexpressed GPX4 to inhibit ferroptosis. This intervention not only successfully suppressed ferroptosis but also led to significant enhancements in cell proliferation, migration, invasion, and the induction of EMT. Therefore, it can be concluded that inhibition of ferroptosis might promote cell migration of GC cells by motivating EMT.

Emerging evidence suggests that the activation of EMT signaling can also increase the susceptibility of tumor cells to ferroptotic cell death. It mentions that factors like Cadherin 1 and integrin subunits α6 and β4 protect against ferroptosis, while the activation of the Hippo pathway (involving YAP1 and WWTR1/TAZ) promotes ferroptosis in cancer cells [19]. These findings highlighted the importance of the EMT pathway as a therapeutic target for sensitizing tumors to ferroptosis-based therapies. Besides, some studies have demonstrated the significance of ferroptosis in the metastasis potential of other types of tumors as well. For example, Ubellacker et al. reported that lymphoid tissue protects cancer cells against ferroptosis, thereby promoting melanoma metastasis [20]. Additionally, in a natural mouse model of HER2-positive breast cancer, initiation of ferroptosis inhibited the spread of the tumor to the brain [21]. Li et al. found that stabilizing GPX4 protein can maintain ROS homeostasis in GC cells during EMT and inhibit ferroptosis, thereby promoting GC metastasis [22]. Thus, based on the results obtained from our investigations in GC cells and the relevant published studies, and considering that patients with distant metastasis are typically not suitable candidates for surgical intervention, we went on to analyze tumor tissues derived from GC patients with or without lymphatic metastasis. Our aim was to shed light on the roles of ferroptosis and EMT in the metastatic process of GC. And the immunohistochemical analysis of tumor tissues from GC patients indicated that lymphatic metastasis was associated with higher potential for ferroptosis inhibition and EMT induction.

Despite the availability of numerous treatment options for GC, the overall curative effect and prognosis are still poor [23]. GPX4 maintains intracellular redox homeostasis by inhibiting lipid peroxidation and shielding cells from ferroptosis [24], and has been found to be closely associated with tumor progression in previous studies [19, 25]. Therefore, we aimed to elucidate the potential role of GPX4 expression in clinical outcomes of GC patients. Although Li et al. demonstrated elevated levels of GPX4 protein in tumor tissues of gastric and colon cancer compared to adjacent normal mucosa tissues [22], and Lu et al. showed that GPX4 inhibition can suppress cell migration and invasion in renal cell carcinoma [26], it is currently unknown whether GPX4 expression correlates with prognosis of GC patients. We utilized Kaplan–Meier survival curves to examine the correlation of overall survival with GPX4 expression in 178 gastric cancer (GC) patients in our study and the findings revealed a significant reduction in overall survival in GC patients with high GPX4 expression.

## Conclusion

**Fig. 9:**
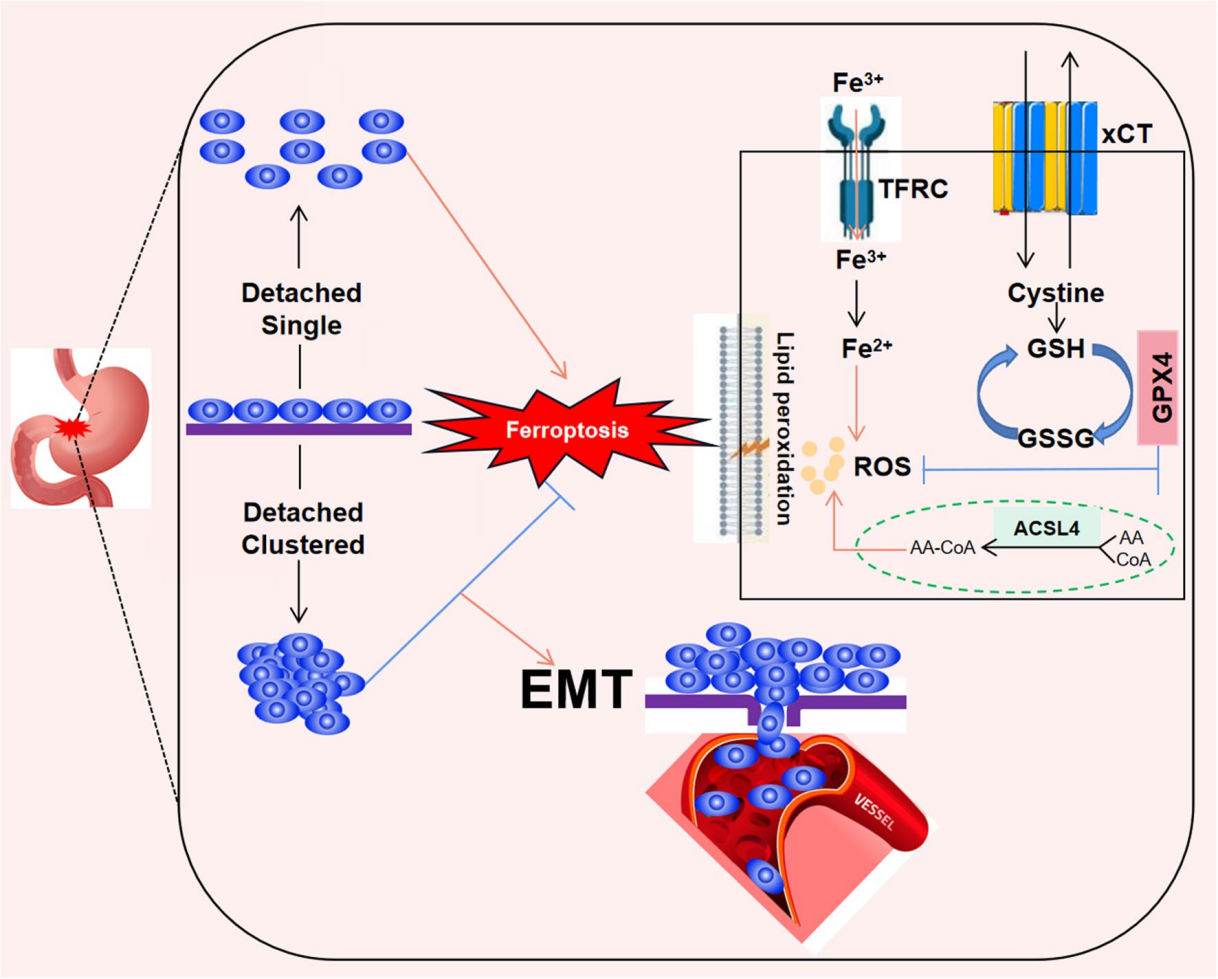
Schematic diagram about the central role of anoikis and ferroptosis in metastatic colonization of gastric cancer. The clustering status of detached gastric cancer cells inhibits anoikis-induced ferroptosis in both GPX4-dependent and GPX4-independent pathway and promotes cancer migration by regulating EMT

In this study, we conducted a comprehensive assessment of the potential role of ferroptosis in gastric cancer (GC) metastasis. In vitro experiments revealed that detached single GC cells are prone to ferroptotic cell death, whereas clustered detached cells are able to evade anoikis-induced ferroptosis and survive in circulation. We further demonstrated that overexpression of the ferroptosis inhibitor GPX4 promotes proliferation, migration, invasion and EMT of GC cells, indicating that ferroptosis inhibition favors metastasis. Immunohistochemical analysis of 54 tumor tissues from GC patients indicated that lymphatic metastasis is associated with higher potential for ferroptosis inhibition and EMT stimulation. Additionally, we analyzed the relationship between overall survival and GPX4 expression in tumor tissues from 178 GC patients. Kaplan–Meier survival curves demonstrated a significant decrease in OS among GC patients with high GPX4 expression, suggesting that GPX4 may be a crucial prognostic factor for GC patients (Fig. 9). Collectively, our findings provide the first evidence that inhibition of ferroptosis plays a vital role in GC metastasis, offering a promising strategy for GC patients with metastatic potential.

### Supplementary Information

**Additional file 1: Figure S1.** Statistical analysis of grayscale scanning of Western Blot bands for three gastric cancer cell lines: AGS, MKN45, and HGC27. The analysis compares the expression levels of ACSL4, TFRC, and HO-1 between the adherent and detached single cell groups. The expression levels of GPX4 and SLC7A11 are compared between the detached single and detached clustered cell groups. Data represent the mean ± SD from more than three independent experiments. Student’s t-test was used to determine statistical significance: *p < 0.05, **p < 0.01.

**Additional file 2: Figure S2.** Evaluation of MDA, GSH, and ROS Levels in Detached Single GC Cells with GPX4 Overexpression and Vector. (A) Intracellular MDA levels. (B) Intracellular GSH levels. (C) The cellular ROS levels were analyzed by a flow cytometer. (*P < 0.05, **P < 0.01, ***P < 0.001).

## Data Availability

The datasets used and/or analyzed during the current study are available from the corresponding author on reasonable request.

## Abbreviations

GC: Gastric cancer
ECM: Extracellular matrix
ROS: Reactive oxygen species
GSH: Glutathione
GPX4: GSH peroxidase 4
CCK8: Cell Counting Kit-8
TEM: Transmission electron microscopy
IHC: Immunohistochemistry
EDTA: Ethylene diamine tetraacetic acid
EMT: Epithelial-mesenchymal transition
OS: Overall survival
