# Supplementary figures and images for: The clustering status of detached gastric cancer cells inhibits anoikis-induced ferroptosis to promote metastatic colonization
Source: Cancer Cell Int. 2024 Feb 18;24:77. doi: 10.1186/s12935-024-03260-1 (PMC10874580; doi:10.1186/s12935-024-03260-1)

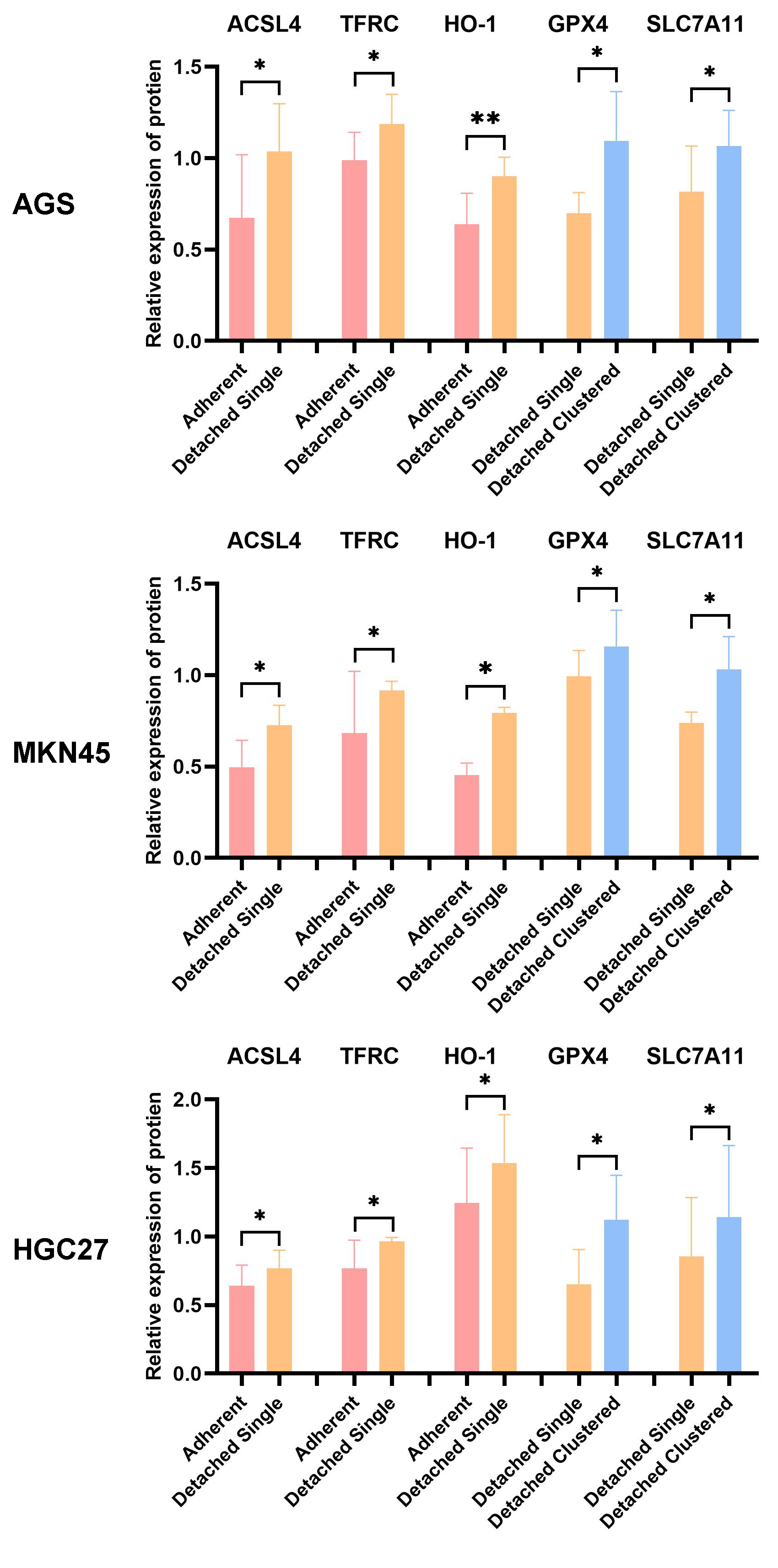

Supplement: Supplementary file 1 — Additional file 1: Figure S1. Statistical analysis of grayscale scanning of Western Blot bands for three gastric cancer cell lines: AGS, MKN45, and HGC27. The analysis compares the expression levels of ACSL4, TFRC, and HO-1 between the adherent and detached single cell groups. The expression levels of GPX4 and SLC7A11 are compared between the detached single and detached clustered cell groups. Data represent the mean ± SD from more than three independent experiments. Student’s t-test was used to determine statistical significance: *p < 0.05, **p < 0.01. [file 12935_2024_3260_MOESM1_ESM.tiff]

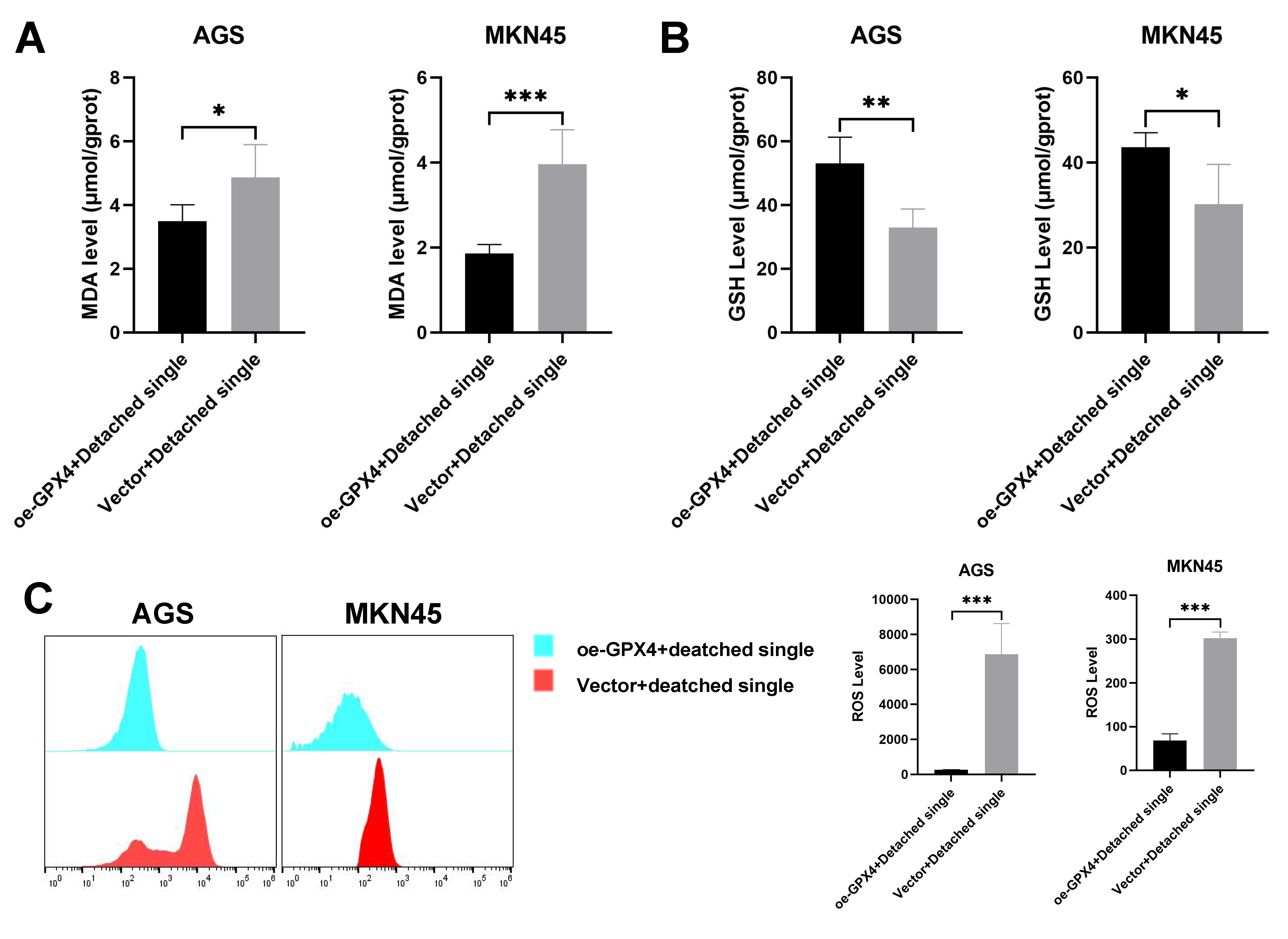

Supplement: Supplementary file 2 — Additional file 2: Figure S2. Evaluation of MDA, GSH, and ROS Levels in Detached Single GC Cells with GPX4 Overexpression and Vector. (A) Intracellular MDA levels. (B) Intracellular GSH levels. (C) The cellular ROS levels were analyzed by a flow cytometer. (*P < 0.05, **P < 0.01, ***P < 0.001). [file 12935_2024_3260_MOESM2_ESM.tiff]
